# Changes in Plant Nitrogen Resorption During Restoration in Inner Mongolia, China

**DOI:** 10.3390/plants15081203

**Published:** 2026-04-15

**Authors:** Xiang Li, Takafumi Miyasaka, Hao Qu

**Affiliations:** 1 Graduate School of Environmental Studies, Nagoya University, Furo-cho, Chikusa-ku, Nagoya-shi 464-8601, Aichi, Japan; lx714x@163.com; 2 Faculty of Environment and Information Studies, Keio University, 5322 Endo, Fujisawa-shi 252-0882, Kanagawa, Japan; 3 Key Laboratory of Ecological Safety and Sustainable Development in Arid Lands, Urat Desert-Grassland Research Station, Northwest Institute of Eco-Environment and Resources, Chinese Academy of Sciences, Lanzhou 730000, China; quhao@lzb.ac.cn

**Keywords:** nitrogen, restoration, functional groups, dryland grasslands

## Abstract

Tree and shrub planting is a widely used strategy to restore degraded semi-arid grasslands. Although nutrient resorption is a key adaptation to nutrient-limited environments, its dynamics at decadal scales remain poorly understood. In this study, we measured species-averaged nitrogen resorption efficiency (NRE) at both community and functional group levels, together with soil nutrients, across 20- and 40-year shrub-planted sites and a 40-year tree-planted site in Inner Mongolia, China. At the community level, green and senesced leaf nitrogen (N) concentrations, NRE, and aboveground biomass did not differ significantly among sites. However, clear differences emerged at the functional group level: Poaceae exhibited higher NRE than forbs and lower senesced leaf N than both forbs and Fabaceae. As restoration progressed, Poaceae replaced forbs as the dominant group, coinciding with increased soil nutrient availability. Notably, NRE in Poaceae declined with increasing soil nutrients, suggesting a shift toward greater reliance on direct soil nutrient uptake. This shift, combined with the production of low-nitrogen litter by dominant Poaceae species, may ultimately slow soil nutrient accumulation. Our findings highlight the importance of functional group dynamics in regulating long-term nutrient resorption and cycling and suggest that managing Poaceae dominance could enhance long-term soil nutrient enrichment and biodiversity in restored semi-arid grasslands.

## 1. Introduction

Overgrazing has accelerated grassland degradation in China, leading to declines in vegetation cover and biomass [1,2,3]. To restore degraded grasslands, the Chinese government has implemented measures such as grazing exclusion and the planting of trees or shrubs [4,5,6,7,8]. Species adapted to arid environments can reduce wind erosion, stabilize sand, and improve soil water retention and soil carbon (C) sequestration [9,10]. In addition, tree and shrub planting increases litter inputs, thereby enhancing soil nutrient availability. These processes can create favorable conditions for plant establishment and promote vegetation and soil recovery, particularly during intermediate stages of restoration in arid and semi-arid ecosystems [11,12,13]. However, over longer timescales (>40 years), afforestation may lead to soil moisture and nutrient depletion, potentially constraining restoration outcomes [14]. Increased competition for the limited soil nutrients may also reduce species diversity [15]. Therefore, a better understanding of long-term restoration effects on ecosystem processes is needed.

Nutrient resorption recycles nutrients from senesced tissues for plant growth and reduces plant dependence on external soil nutrient supply [16], a crucial nutrient conservation strategy for plants [17]. This is also a key ecosystem nutrient cycling process because it determines litter quality and decomposition by controlling litter nutrient content [18]. Previous studies have identified both biotic and abiotic controls on nutrient resorption, including soil nutrient availability [18,19,20] and leaf nutrient concentrations [21,22]. Some studies have found that plants growing in nutrient-poor environments have higher resorption than in fertile environments [18,19,20], while others have reported no relationship between soil nutrient availability and nutrient resorption [23,24]. Similarly, while nitrogen (N) and phosphorus (P) resorption often decreases with increased leaf N and P concentrations across environmental gradients [21,22], other studies have found no such effect [25]. Because shrub and tree planting can substantially alter both plant and soil nutrient status in drylands [7,26], examining how nutrient resorption responds to restoration is essential for understanding ecosystem nutrient dynamics.

Plant functional group, which share similar traits such as life history, morphology, and resource-use strategies, often exhibit comparable responses to environmental change [27,28,29]. Nutrient resorption, however, can vary significantly among functional groups. For example, N resorption efficiency (NRE) is often higher in Poaceae than in forbs, potentially reflecting greater nutrient demand [30,31]. During restoration, changes in species composition and soil nutrient availability can alter competition intensity and resource allocation, leading to shifts in nutrient acquisition strategies [32,33]. Despite growing interest in functional group dynamics, how nutrient resorption changes along long-term restoration gradients remains unclear.

N is widely considered a limiting nutrient for plant productivity in northern China [34]. Leaf N is closely related to photosynthesis, litter decomposition, ecosystem productivity, and enzymatic activity [35,36]. In this study, we investigated leaf N concentrations and their resorption at the functional group level across two shrub-planted sites (*Caragana microphylla*; 20 and 40 years), and one tree-planted site (*Populus simonii*; 40 years) in the Horqin Sandy Land, all under grazing exclusion. The objectives were to (1) quantify changes in N resorption among plant functional groups during long-term restoration, and (2) examine relationships between N resorption and plant and soil properties. We hypothesized that: (i) increasing soil nitrogen availability with restoration would reduce overall plant NRE, and (ii) NRE responses would differ among plant functional groups due to variation in nitrogen-use strategies. Our findings provide insights into plant internal nitrogen cycling and inform management of restored semi-arid grasslands.

## 2. Results

### 2.1. Soil Properties at Different Restoration Sites

Soil organic carbon (SOC), total nitrogen (STN), and total phosphorus (STP) were significantly higher in PO40 than in CA20 (Table 1). Soil water content (WC) in PO40 was significantly higher than in CA40, and WC in CA40 was significantly higher than in CA20. Soil ammonium nitrogen (NH_4_^+^) did not change significantly at the three sites. Soil nitrate nitrogen (NO_3_^−^) in CA40 was significantly lower than in CA20 and PO40.

### 2.2. Plant Properties in Different Restoration Sites

**Figure 1:**
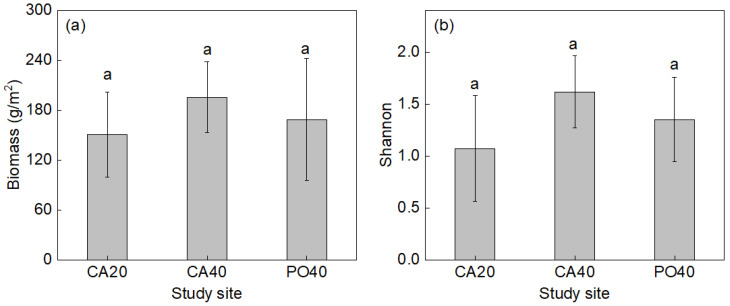
Aboveground biomass (**a**), Shannon’s diversity index (**b**) in three sites with standard error (SE) bar. Identical letters indicate no significant differences (*p* > 0.05) between the three sites.

**Figure 2:**
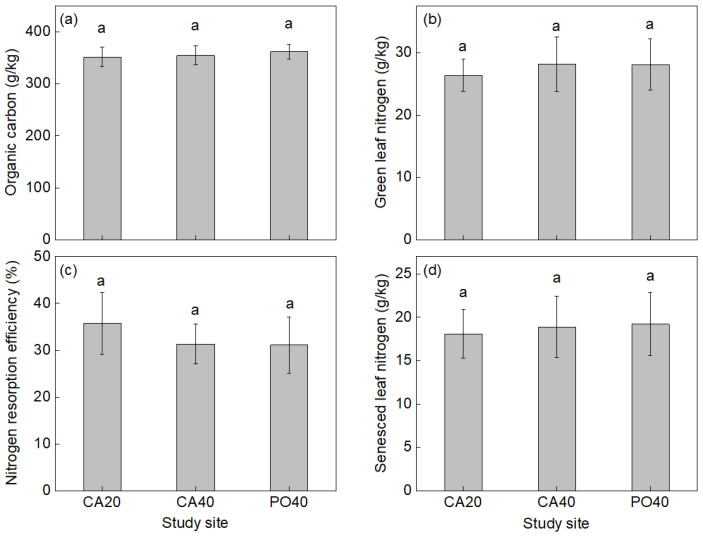
Organic carbon (**a**), green leaf nitrogen concentrations (**b**), nitrogen resorption efficiency (**c**), senesced leaf nitrogen concentrations (**d**) in three sites with standard error (SE) bar. Identical letters indicate no significant differences (*p* > 0.05) between the three sites.

At the plant community level, there were no significant differences in aboveground biomass and Shannon’s diversity index among the three sites (*p* > 0.05; Figure 1). Similarly, leaf nutrient traits, including C in green leaves, N in green and senesced leaves, and NRE, remained stable across all sites (*p* > 0.05; Figure 2).

**Figure 3:**
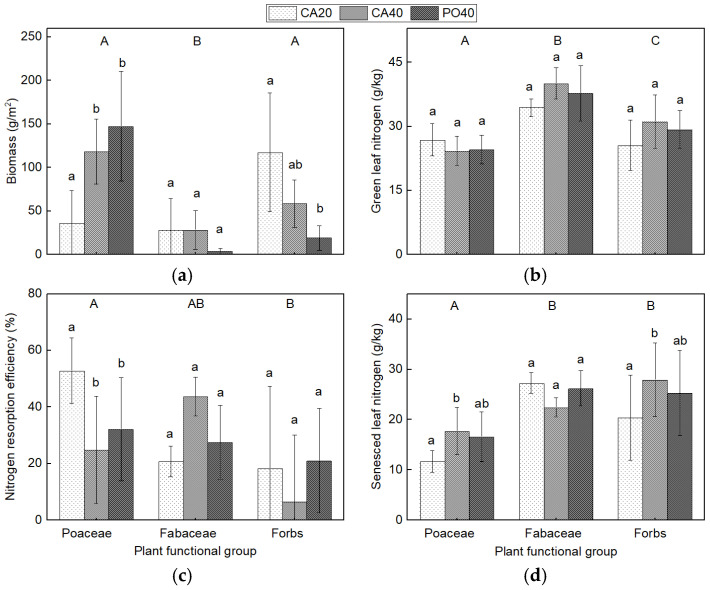
Aboveground biomass (**a**), green leaf nitrogen concentrations (**b**), nitrogen resorption efficiency (**c**), and senesced leaf nitrogen concentrations (**d**) at three sites. Different lowercase letters indicate significant differences (*p* < 0.05) among grasslands for a given plant functional group. Different uppercase letters indicate significant differences (*p* < 0.05) among plant functional groups.

In contrast, at the plant functional group level, Fabaceae had the lowest biomass and the highest N in green leaves (Figure 3). Poaceae showed significantly higher NRE than forbs, and their senesced leaf N was significantly lower than that of both Fabaceae and forbs. Within the same functional group across different sites, the biomass of Poaceae was significantly higher in PO40 and CA40 than in CA20, while the biomass of forbs was significantly lower in PO40 compared to CA20. Poaceae NRE in CA20 was significantly higher than in both CA40 and PO40. The N content of senesced Poaceae and forb leaves in CA20 was significantly lower than in CA40.

### 2.3. The Relationship Between Plant and Soil Factors and Species Composition

**Figure 4:**
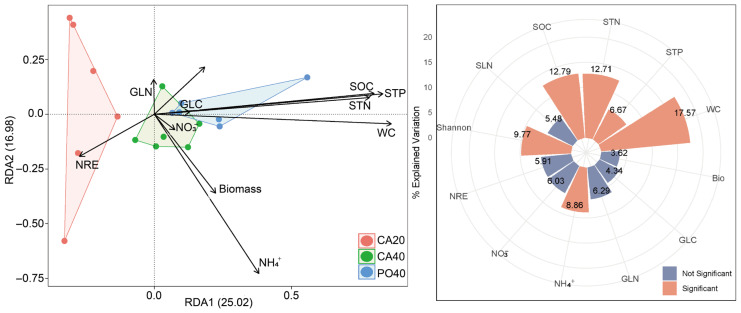
Redundancy analysis (RDA) and hierarchical partitioning (HP) analysis showing the relationship between species composition, plant and soil variables, and contribution of plant and soil variables to species composition. Bio, aboveground biomass; GLC, carbon in green leaves; GLN, nitrogen in green leaves; SLN, nitrogen in senesced leaves; NRE, nitrogen resorption efficiency; SOC, soil organic carbon; STN, soil total nitrogen; and STP, soil total phosphorus; WC, soil water content; NH_4_^+^, ammonium nitrogen; NO_3_^−^, nitrate nitrogen.

Redundancy analysis showed that the soil and plant properties (SOC, STN, STP, NH_4_^+^, NO_3_^−^, plant aboveground biomass, NRE, and N in green and senesced leaves) explained 42% of the total variation in plant community, with axes 1 and 2 explaining 25.02% and 16.98% of the total variation, respectively (Figure 4). Axis 1 was positively correlated with SOC, STN, STP, and WC and negatively correlated with NRE. Along the first axis, the three sites were ordered from highest to lowest as PO40, CA40 and CA20, reflecting differences in soil nutrient levels. Hierarchical partitioning analysis showed that WC (17.57%), SOC (12.79%), and STN (12.71%) contributed most strongly to the variation, followed by Shannon diversity index (9.77%), NH_4_^+^ (8.86%), and STP (6.67%).

The biomass and senesced leaf N of Poaceae were significantly positively correlated with soil C, N, P, and WC, while NRE of Poaceae was significantly negatively correlated with WC (Table 2). For Fabaceae, NH_4_^+^ and NO_3_^−^ were significantly correlated positively and negatively with Fabaceae biomass and NRE, respectively. Green leaf N and NRE in Fabaceae were significantly positively correlated with WC. The biomass of forbs was negatively correlated with WC. Forbs showed a significant negative correlation between biomass and WC. All other correlations were not statistically significant.

## 3. Discussion

### 3.1. N Resorption Response to Restoration

At the community level, plant variables did not change significantly among sites. Previous studies have shown that vegetation density, cover, and diversity can stabilize within several years after shrub planting in the Horqin Sandy Land [8]. In the present study, all sites had been maintained for more than 20 years, which could explain the stability of aboveground biomass, diversity, leaf nutrient concentrations, and N resorption at the community level.

Despite this stability, species composition differed markedly among sites. Specifically, the dominant species shifted from forbs to Poaceae along the restoration gradient. This pattern is consistent with mechanisms reported in previous studies, such as increased light limitation associated with greater plant height [37] and reduced resource acquisition by shallow-rooted species [38,39]. However, as light availability and root traits were not directly measured here, these explanations should be interpreted with caution. Previous studies have reported that overgrazing leads to a decline in Poaceae cover and biomass, while the cover and biomass of forbs often increase [40,41,42,43]. In the present study, the exclusion of grazing and the recovery of soil nutrient availability may also have contributed to the increase in Poaceae biomass. Together, these results suggest that while community-level traits may stabilize relatively early, species composition can continue to shift over longer restoration timescales.

The absence of significant changes in community-level NRE likely reflects compensatory dynamics among functional groups. Although NRE in Poaceae decreased with restoration, their increasing biomass—combined with the replacement of forbs, which typically exhibit lower NRE—appears to buffer changes at the community level.

At the functional group level, Poaceae biomass increased with restoration and was associated with lower green leaf N concentrations compared with forbs and Fabaceae, indicating higher nitrogen-use efficiency [44,45]. In addition, NRE was higher in Poaceae than in forbs, consistent with their greater nutrient demand [30,31]. In contrast, forbs, which generally have lower nutrient demand and shallow root systems, exhibited lower NRE [31]. However, previous work in Inner Mongolia reported a different pattern, with NRE highest in forbs, followed by Poaceae and then N-fixing species [46]. Although environmental conditions are similar, the restored nature of the sites in the present study likely alters interspecific competition, which may explain these differences.

NRE in Poaceae was higher in CA20 than in CA40 and PO40, likely reflecting stronger nutrient limitation [44] and competition with forbs earlier restoration stages. As soil nutrient availability increased, Poaceae appeared to shift toward greater reliance on direct nutrient uptake. In contrast, NRE in forbs did not change significantly across sites, which may indicate a compensatory response to increasing competition from Poaceae.

### 3.2. Relationships Between N Resorption, Plant, and Soil Variables

Many studies have reported that increased soil nutrient availability leads to reduced nutrient resorption [19,47,48], as plants can acquire nutrients more readily from the soil. In the present study, however, Poaceae and Fabaceae showed contrasting patterns in NRE and senesced leaf N in relation to soil C, N, P, and WC, suggesting distinct nutrient-use strategies among functional groups.

For Poaceae, the positive relationship between senesced leaf N and soil nutrients indicates that litter nutrient content may be more responsive to soil conditions than resorption efficiency is [49]. Litter quality is a key driver of decomposition [50] and is closely associated with soil nutrient input [21]. Consistent with this, we found that Poaceae biomass increased alongside increased soil nutrient availability, suggesting that increased Poaceae litter serves as a primary nutrient source. Nonetheless, the dominance of Poaceae—characterized by low litter N concentrations—may ultimately slow soil nutrient accumulation and intensify nutrient limitation. These findings suggest that management practices, such as mowing, may be necessary to regulate Poaceae dominance and support long-term soil nutrient enrichment in semi-arid grasslands.

Redundancy analysis indicated that soil properties explained a substantial proportion of the variation in species composition along the restoration gradient. In semi-arid grasslands, water and nutrient limitations are closely linked [51], and improvements in soil water content are a major driver of aboveground productivity [52]. Although community-level biomass and diversity remained stable across sites, species composition continued to shift, largely in response to differences in soil conditions associated with restoration age.

The stronger influence of NH_4_^+^ compared to NO_3_^−^ on species composition suggests a preference for ammonium uptake, which is consistent with previous studies [53,54]. In arid environments, limited nitrification reduces NO_3_^−^ availability, and NH_4_^+^ can be assimilated with lower energy costs [55], making it a more efficient nitrogen source under water-limited conditions. In addition, the observed negative relationships between plant NRE and soil C, N, P, and water content are consistent with established patterns in the literature [16].

## 4. Materials and Methods

### 4.1. Study Area

The study was carried out in central Naiman County, Inner Mongolia, China (42°55′ N, 120°42′ E, 360 m elevation), which is in a temperate, continental semi-arid monsoon zone. The mean annual temperature is 6.4 °C, ranging from −13.1 °C in January to 23.7 °C in July. The mean annual precipitation is 360 mm, 70% of which occurs during the growing season from May to August. The primary vegetation is mixed meadow and sandy steppe, typically populated by native plants, including grasses (e.g., *Setaria viridis, Digitaria ciliaris*, and *Cleistogenes squarrosa*), forbs (e.g., *Artemisia scoparia*, *Chenopodium acuminatum*, and *Salsola collina*), and shrubs (e.g., *Artemisia halodendron*). The zonal soil is classified as sandy chestnut soil with a coarse texture and loose structure, which is mostly equivalent to the Orthi-Sandic Entisols of sand origin in terms of the FAO-UNESCO system [56]. We selected three sites: one planted with trees (*P*. *simonii*) for 40 years, and two others planted with shrubs (*C*. *microphylla*) for 20 and 40 years, respectively. All sites have been fenced. These sites provided us with the opportunity to observe long-term restoration.

### 4.2. Field Survey and Sampling

This study employed a chronosequence (space-for-time substitution) design to evaluate vegetation and soil dynamics under different restoration durations. Three sites, all planted and protected from grazing, were selected in August 2024 for the present study: CA20 (*C*. *microphylla*, 20 years, 42°50′3″ N 120°41′1″ E), CA40 (*C*. *microphylla*, 40 years, 42°57′5″ N 120°46′6″ E) and PO40 (*P*. *simonii*, 40 years, 42°57′ N 120°42′13″ E). All sites were classified as shifting sand dunes when restoration commenced. At each site, six paired quadrats were randomly established (totaling 18 plots): one 0.5 × 0.5 m quadrat for clipping all above-ground biomass at the soil surface in early August, and an adjacent 1 × 1 m quadrat for leaf collection. For leaf sampling, 10 to 20 fully mature green leaves of similar size were randomly selected for each species during the same period in early August, and senesced leaves were collected from the same quadrats following the same protocol in early October. Plant species were classified into three plant functional groups (Poaceae, Fabaceae, and forbs). Fabaceae were classified separately from other forbs. Because their nitrogen acquisition is characterized by symbiotic associations with *Rhizobium*, their nutrient strategy fundamentally differs from that of strictly soil-dependent forbs. All leaves were oven-dried at 70 °C for 48 h to a constant weight for the determination of organic C and total N concentrations. C was quantified using the external heating method with titration according to the Chinese National Standard GB 7857-87 [57]. N was measured using the Kjeldahl digestion method according to NY/T 1121.24–2012 [58], using a Kjeldahl nitrogen analyzer (Hanon Instruments, Jinan, China).

After collecting the clipped plants, three soil cores 0–10 cm in depth [59,60,61] were collected with an auger (5 cm in diameter) from each quadrat and then mixed into one composite sample. These soil samples were divided into three subsamples: (1) oven-dried to a constant weight at 105 °C to determine WC; (2) air-dried, ground, and passed through a 2 mm sieve for measurements of SOC, STN, and STP; and (3) kept fresh and passed through a 2 mm sieve for the determination of soil NO_3_^−^ and NH_4_^+^. SOC and STN were determined using the same methods as the leaf analysis. STP was measured using the molybdenum–antimony colorimetric method with an i5 UV-Vis spectrophotometer (Lambda 25 Perkin Elmer, Shelton, CT, USA). NO_3_^−^ and NH_4_^+^ were quantified by a continuous flow analyzer (AA3, SEAL Analytical, Norderstedt, Germany) after the samples were extracted with a KCl solution. All nutrient concentrations, biomass, and other measurements are expressed on a dry-mass basis.

### 4.3. Data Analysis

N concentrations in green and senesced leaves were used to calculate nitrogen resorption efficiency [62] for each species, which was calculated as follows:

(1) $$NRE=\frac{N_{green}-N_{senesced}}{N_{green}}\times 100\%$$

where N_green_ and N_senesced_, represent N concentration in the green leaves and senesced leaves, respectively. At the functional group and community levels, NRE for each quadrat was computed as the unweighted mean of the species-level NRE values.

Shannon’s diversity index [63] was calculated as follows:

(2) $$Shannon’s\;diversity\;index\left(H\right):H=-\sum_{i=1}^{n}\left(P_{i}\ln P_{i}\right)$$

where: P_i_ is the relative abundance of species i based on its biomass recorded on each quadrat at the site. Relative abundance was calculated using biomass rather than individual counts to more accurately represent species’ ecological dominance, which is particularly relevant in grassland systems with highly variable plant sizes [64].

One-way ANOVA was first conducted to test for differences among the three grassland sites in terms of plant nutrient concentrations, NRE, aboveground biomass (of plant functional groups and communities), and soil properties. Tukey’s multiple comparison test was then applied to identify pairwise differences. Redundancy analysis (RDA) was performed to examine the relationships between plant status, soil nutrients, and plant communities. To quantitatively assess the factors driving community changes, a hierarchical partitioning (HP) analysis was conducted. HP provides a robust approach for addressing collinearity, as it decomposes the total variation across all subsets of variables. This allows the quantification of the independent and shared contributions of each variable based on all possible model combinations [65,66]. The Pearson correlation coefficient was calculated to assess the relationships between soil properties, plant nutrient concentrations, and NRE, in different functional groups. All analyses were performed using R version 4.3.1 [67].

## 5. Conclusions

This study shows that long-term restoration in semi-arid grasslands alters species composition and plant nutrient cycling. Contrary to our initial hypothesis, community-level NRE remained stable despite increased soil nutrient availability. Instead, NRE responses differed among plant functional groups.

Poaceae increasingly dominated with restoration, and their high biomass and low-nitrogen litter were associated with changes in soil nutrient dynamics. In particular, the observed decline in NRE in Poaceae (contrasting with stable NRE in forbs and Fabaceae) suggests a shift toward a greater reliance on direct soil nutrient uptake. Over time, the dominance of Poaceae may slow soil nutrient accumulation due to the low nutrient quality of their litter.

Although this study is based on a single-year sampling and requires further investigation into seasonal or interannual variability, the results highlight the importance of functional group dynamics in regulating nutrient cycling. These findings suggest that managing dominant species, for example through mowing, may help balance productivity, nutrient retention, and biodiversity, thereby supporting long-term restoration in semi-arid grasslands.

## Figures and Tables

**Table 1:** Soil properties at 0–10 cm in depth across sampling sites in the Horqin Sandy Land.

Soil Property	Units	Site		
		CA20	CA40	PO40
SOC	g/100 g	0.28 ± 0.15 a	0.44 ± 0.08 ab	0.73 ± 0.36 b
STN	g/100 g	0.04 ± 0.02 a	0.06 ± 0.01 ab	0.09 ± 0.04 b
STP	mg/100 g	0.09 ± 0.02 a	0.13 ± 0.02 a	0.20 ± 0.07 b
WC	%	2.68 ± 1.06 a	7.37 ± 1.32 b	11.26 ± 4.49 c
NH_4_^+^	mg/kg	3.51 ± 0.22 a	3.69 ± 0.12 a	3.61 ± 0.16 a
NO_3_^−^	mg/kg	12.73 ± 6.57 a	3.95 ± 1.99 b	14.36 ± 4.29 a

CA20, shrub-planted sites maintained for 20 years; CA40, shrub-planted sites maintained for 40 years; PO40, tree-planted sites maintained for 40 years; SOC, soil organic carbon; STN, soil total nitrogen; and STP, soil total phosphorus; WC, soil water content; NH_4_^+^ ammonium nitrogen; NO_3_^−^, nitrate nitrogen. Values with different letters within columns are significantly different at *p* < 0.01. All data are presented as the mean values ± standard error (SE).

**Table 2:** Pearson correlation coefficients of leaf nutrient concentrations, N resorption efficiencies, and aboveground biomass with various soil properties.

Functional Group		SOC	STN	STP	WC	NH_4_^+^	NO_3_^−^
Forbs	GLC	−0.05	−0.07	−0.06	0.00	−0.11	−0.05
	GLN	0.25	0.27	0.2	0.31	0.24	−0.34
	NRE	−0.06	−0.07	−0.13	−0.02	0.41	−0.12
	SLN	0.20	0.22	0.29	0.28	−0.17	−0.24
	Biomass	−0.39	−0.39	−0.42	−0.49 *	−0.20	−0.04
Poaceae	GLC	0.21	0.22	0.23	0.40	0.41	0.35
	GLN	0.15	0.17	0.11	0.04	−0.13	−0.11
	NRE	−0.39	−0.39	−0.42	−0.61 **	−0.47	0.30
	SLN	0.51 *	0.53 *	0.51 *	0.67 **	0.42	−0.31
	Biomass	0.69 **	0.68 **	0.67 **	0.75 **	0.43	0.10
Fabaceae	GLC	−0.47	−0.44	−0.52 *	−0.51	0.11	−0.21
	GLN	0.41	0.44	0.42	0.61 *	0.39	−0.48
	NRE	0.38	0.41	0.54	0.66 **	0.19	−0.69 **
	SLN	−0.43	−0.41	−0.48	−0.52	−0.03	0.51
	Biomass	−0.38	−0.33	−0.33	−0.26	0.57 *	−0.23

Results are presented separately for different plant functional groups and the entire community. * and ** indicate significant correlations at *p* < 0.05 and *p* < 0.01, respectively. GLC, carbon in green leaves; GLN, nitrogen in green leaves; SLN, nitrogen in senesced leaves; NRE, nitrogen resorption efficiency; SOC, soil organic carbon; STN, soil total nitrogen; STP, soil total phosphorus; WC, soil water content; NH_4_^+^ ammonium nitrogen; NO_3_^−^, nitrate nitrogen.

## Data Availability

The data are available in Zenodo at https://doi.org/10.5281/zenodo.18015450.

## Abbreviations

The following abbreviations are used in this manuscript:

CA20	Shrub planting sites maintained for 20 years
CA40	Shrub planting sites maintained for 40 years
PO40	Tree planting sites maintained for 40 years
SOC	Soil organic carbon
STN	Soil total nitrogen
STP	Soil total phosphorus
WC	Soil water content
NH_4_^+^	Ammonium nitrogen
NO_3_^−^	Nitrate nitrogen
GLC	Carbon in green leaves
GLN	Nitrogen in green leaves
SLN	Nitrogen in senesced leaves
NRE	Nitrogen resorption efficiency
